# Effects of Continuous Theta Burst Stimulation on Behavior and NMDA Receptor Subunits in the Trimethyltin-Induced Alzheimer’s-like Disease Model

**DOI:** 10.3390/biomedicines14020391

**Published:** 2026-02-08

**Authors:** Marina Zaric Kontic, Milica Zeljkovic Jovanovic, Andjela Stekic, Jelena Stanojevic, Ivana Stevanovic, Dejan Stevic, Milica Ninkovic, Milorad Dragic

**Affiliations:** 1 Department of Molecular Biology and Endocrinology, Vinca Institute of Nuclear Sciences—National Institute of the Republic of Serbia, University of Belgrade, Mike Petrovica Alasa, 11351 Belgrade, Serbia; 2 Center for Translational Neurosciences, Faculty of Biology, University of Belgrade, 11158 Belgrade, Serbia; 3 Medical Faculty of Military Medical Academy, University of Defence, 11040 Belgrade, Serbia

**Keywords:** repetitive transcranial magnetic stimulation, continuous theta burst stimulation, anxiety-like behavior, learning and memory, trimethyltin-induced neurodegeneration, NMDA receptors, hippocampus, Alzheimer’s disease animal model

## Abstract

**Background**: Trimethyltin (TMT)-induced neurodegeneration leads to molecular and behavioral changes resembling those of Alzheimer’s disease (AD), making it a relevant model for investigating potential therapeutic interventions. Continuous theta burst stimulation (cTBS) has shown promise in psychiatric and neurological disorders but remains largely unexplored in AD models. **Methods**: Adult male Wistar rats were divided into four experimental groups: intact, TMT (8 mg/kg, ip) and TMT treated with cTBS or a sham protocol for three weeks. The open field test and novel object recognition test were used to assess anxiety-like behavior, memory, and learning, respectively. The extent of microgliosis in the hippocampus was assessed by immunohistochemistry, while protein expression was estimated by Western blot. **Results**: cTBS improved TMT-mediated changes in anxiety-like behavior, learning, and memory and reduced microgliosis in the CA1 hippocampal region. Both TMT and cTBS affected NMDAR subunits, with the most significant finding being a cTBS-mediated decrease in NR2B, which was previously increased by TMT. **Conclusions**: These are the first data on the beneficial effects of cTBS on behavioral and molecular changes in a model of neurodegeneration that mimics some of the key aspects of AD pathology. Further research is needed to clarify the therapeutic potential of cTBS in AD treatment.

## 1. Introduction

Neurodegenerative disorders affect millions of people worldwide and are characterized by neurodegeneration, a complex pathological cascade that leads to progressive deterioration of neuronal function, degeneration of synapses and axons, and ultimately neuronal death [1]. Alzheimer’s disease (AD) is a progressive and one of the most prevalent neurodegenerative diseases [2,3], associated with striking cognitive and behavioral impairments [4] and distinct molecular and cellular alterations, including extracellular amyloid peptide plaques, hyperphosphorylated Tau protein fibrillary aggregation [5,6], neuronal death [5], synaptic changes and damage [7], mitochondrial abnormalities [8], and neuroinflammation [9,10]. Glutamatergic dysfunction, particularly alterations of the N-Methyl-D-aspartate receptor (NMDAR) and associated excitotoxicity, has been described as one of the most prominent features of AD [11,12].

Animal models have long been indispensable for studying the etiology and pathological mechanisms of neurodegenerative diseases such as AD and for developing and validating therapeutic strategies [13]. Neurodegeneration induced by trimethyltin (TMT), a potent neurotoxin, has been proposed as a useful model of AD suitable for testing therapeutic approaches because it elicits molecular, cellular, and behavioral outcomes that resemble the major neurological and histopathological features of AD [14,15,16,17,18]. A single intraperitoneal injection of TMT induces a range of symptoms in rats, including seizures, hyperactivity, hyperexcitability, aggression, and severe cognitive deficits [16,17,19,20,21,22]. These manifestations are primarily due to the neuronal death of CA1/CA3 pyramidal neurons in the hippocampus. Damage begins 2–4 days after exposure and worsens over the next 3 weeks, by which time almost all medial and proximal CA3 and CA1 neurons are degenerated [23,24]. Alzheimer’s disease often begins with the destruction of neurons and their connections in the hippocampus and entorhinal cortex [25], so the neurodegeneration described above is a common feature of both the presented TMT model and AD. In addition, TMT triggers a deleterious neurodegenerative cascade involving several behavioral and molecular disturbances, such as learning and memory deficits [16,26,27,28], increased depressive and anxiety-like behavior [16,29], neuroinflammation [24], glutamate-mediated excitotoxicity and intracellular calcium overload [17,18], altered oxidative and nitrosative status [30,31], mitochondrial dysfunction [30,32], and gene expression associated with apoptosis and necrosis [17,32], all of which are seen in AD [5].

Transcranial magnetic stimulation (TMS) is a non-invasive brain stimulation technique in which the TMS device generates an electric current through a plastic-coated coil wire placed over the patient’s or animal’s scalp [33], creating a magnetic field over the cranial tissue and electrical stimulation in the targeted area(s) of the brain [34]. TMS can be applied as a single or repeated pulse. Compared to single-pulse TMS, repetitive TMS (rTMS) modulates cortical activity and promotes after-effects beyond the stimulation period. Different rTMS protocols produce varying after-effects in the brain, with inhibitory effects at low-frequency stimulation (≤1 Hz) and excitatory effects at high-frequency stimulation (≥5 Hz) [35]. rTMS has been used as a research technique and is also an FDA-approved treatment for several psychiatric disorders, as well as a candidate therapy for several neurological disorders [17,36]. Potential beneficial effects of various rTMS protocols on AD symptoms have been reported [33,36,37]. The theta burst stimulation (TBS) protocol is a highly effective version of rTMS that offers a short stimulation time, low stimulus intensity, and improved reliability [26]. The specific rTMS variant, continuous theta burst stimulation (cTBS), produces rapid and long-lasting changes in cortical excitability by inducing durable effects on long-term depression (LTD) [36,37]. To date, clinical research has shown promising effects of cTBS in the treatment of stroke, obsessive–compulsive disorder, major unipolar depression, and drug-resistant epilepsy [37,38,39]. Despite this, cTBS remains unfairly neglected, as most available data relate to the effects of the opposite, excitatory TBS protocol, intermittent theta burst stimulation (iTBS) [16,40]. To the best of our knowledge, there are no available data on the effects of cTBS in AD patients or Alzheimer’s-like disease models.

Considering the hyperactivity and hyperexcitability closely related to TMT-induced neurodegeneration [17] and the LTD-promoting effects of cTBS [36], this specific paradigm was chosen as the therapeutic approach in this study. The uncertainty regarding cTBS as a treatment for neurodegenerative diseases such as AD provided additional scientific motivation to investigate its effects in a model of TMT-induced neurodegeneration. The effects of cTBS on anxiety-like behavior and learning/memory were investigated, as these disturbances are prominent in several neurodegenerative diseases, including AD [41,42]. Because NMDARs play an essential role in generating the long-lasting after-effects of cTBS [36] and are major contributors to both the pathophysiology of AD [1,12] and the behavioral and cognitive traits [43,44] mentioned above, the authors further examined the protein expression of NMDAR subunits as well as vesicular glutamate transporter 1 (vGlut1) in the hippocampus of male Wistar rats.

## 2. Materials and Methods

### 2.1. Animal Housing and Ethical Considerations

A total of 48 two-month-old male Wistar rats (260 ± 20 g), housed at the Center of Veterinary Services animal facility, Ministry of Defence, were used in this study. Animals (3/cage) were maintained under standard conditions (23 ± 2 °C, 12 h light/dark cycle) and were fed standard chow and tap water ad libitum. All experimental procedures were performed in accordance with EU Directive 2010/63 and approved by the Ethics Committee for the Use of Laboratory Animals of the Vinca Institute of Nuclear Sciences—National Institute of the Republic of Serbia, University of Belgrade, Belgrade, Republic of Serbia (authorization number: 323-07-02057/2017-05, issue date: 28 March 2017).

### 2.2. Trimethyltin Intoxication and Experimental Groups

The animals were randomly assigned to four experimental groups: control *(n* = 12), TMT (*n* = 12), TMT + cTBS (*n* = 12), and Sham cTBS (*n* = 12). On day 0, animals in the TMT, TMT + cTBS, and Sham cTBS groups received a single intraperitoneal injection of TMT (8 mg/kg, administered in 1 mL of 0.9% saline), while animals in the control group received the same volume of 0.9% saline. The sham cTBS group was exposed to the noise artifact of cTBS. Specifically, a cage containing two animals was placed next to the MagStim Rapid2 device, and the rats were allowed to hear the sound of stimulation. The animals were then handled in the same way as in the TMT+cTBS group to exclude possible effects of handling, TMS noise, and related factors. The inclusion of the sham cTBS group exposed to 0.9% saline and handling/noise conditions was based on our previously published work using a similar rTMS setup [16,40], where these conditions were shown to have no behavioral or molecular effects. Therefore, these sham conditions are not expected to influence the measured outcomes in the present study. The TMT + cTBS group was treated with cTBS for 3 weeks according to the protocol described in detail below. After three weeks, all animals were killed by decapitation (Harvard Apparatus, Holliston, MA, USA).

### 2.3. Continuous Theta Burst Stimulation Protocol

Three days after intoxication, cTBS was performed as previously described [40]. Briefly, stimulation was delivered using the MagStim Rapid2 device via a 25 mm figure-of-eight coil (MagStim Company, Whitland, UK). The cTBS protocol consisted of a single 40 s train of bursts at a frequency of 5 Hz, each containing 600 pulses. Stimulation intensity was set at 33% of maximum output, just below the motor threshold. The motor threshold was defined as the stimulus intensity that induces a minimal visible motor response in treated animals, most commonly observed as repetitive movement of the mandible muscles resembling chewing. Stimulation was applied by holding the center of the coil just above the anterior skull bone in close contact with the scalp, while the animals were gently held during stimulation. Because the coil is larger than the animal’s skull, application over the anterior skull bone provides evenly distributed stimulation to the entire brain.

### 2.4. Behavioral Tests

All behavioral tests were performed in a secluded room. Before each test, animals were habituated to the room for 60 min. Odors were removed by cleaning the apparatus with 70% alcohol after each animal. After the assessment of behavioral tests, two animals from each group were excluded from further analyses due to the poor or aberrant behavioral scores, and their results were removed from the statistical analysis, as shown in the results presented below.

#### 2.4.1. Open Field Test

After completion of cTBS treatment, an open field test was conducted to assess anxiety-like behavior and general locomotion of the animals. Animals were placed in the center of a black arena (100 × 100 × 50 cm), and their activities were recorded for 5 min and analyzed by qualified, unbiased investigators.

#### 2.4.2. Novel Object Recognition Test

A novel object recognition test (NORT) was used to assess learning and memory performance following cTBS treatment. The test was performed using a modified method described by Ennaceur and Delacour [45]. Animals were placed in the center of the arena, equidistant from two identical objects, and allowed to freely explore the arena and the objects for 5 min (sampling phase). The objects used did not resemble living stimuli, were sufficiently heavy to prevent the rats from moving them, and were tall enough to prevent climbing. After one hour, the animals were returned to the arena in the same position, but a new conical object replaced one of the original objects. Their activity was recorded for 5 min (test phase). Only animals that spent more than 2 s sniffing, climbing, and exploring the objects were included in the analysis. Performance was analyzed by qualified, unbiased investigators. A recognition index (RI) was used to assess exploration efficiency of the novel object, calculated as follows: RI = (Tn − Tf)/(Tf + Tn), where Tn represents the time spent exploring the novel, and Tf represents the time spent exploring the familiar object. A higher RI indicates better learning and memory performance [46].

### 2.5. Immunohistochemistry and Light Microscopy

Following decapitation, brains were rapidly removed from the skull (*n* = 4 *per* group), fixed in 4% paraformaldehyde for 24 h, and cryoprotected in graded sucrose solutions (10%, 20% and 30% in 0.2 M phosphate buffer). Coronal sections (25 μm) were cut, air-dried, and stored at −20 °C. For immunohistochemical analyses, the cryosections were incubated in 0.1% H_2_O_2_ in methanol for 20 min at room temperature, washed 2 × 5 min in PBS, and blocked with 5% normal donkey serum in PBS for 1 h at room temperature. Slides were then incubated with Iba1 antibody (Table 1) at 4 °C overnight. The next day, slides were washed 3 × 5 min in PBS and incubated with the appropriate HRP-conjugated secondary antibody (Table 1). The reaction was visualized with HRP chromogen, 3,3′-S-diaminobenzidine-tetrahydrochloride (DAB, Abcam, Cambridge, UK). The reaction was stopped with PBS, followed by dehydration in graded ethanol (70%, 95% and 100%), and cleared in xylene 2 × 5 min. Sections were mounted with DPX medium (Sigma Aldrich, Saint Louis, MO, USA) and left to dry overnight. Sections were analyzed on a LEITZ DM RB light microscope (Leica Mikroskopie & Systems GmbH, Wetzlar, Germany) under ×50 and ×200 magnifications, and digital images were captured using a LEICA DFC320 CCD camera (Leica Microsystems Ltd., Heerbrugg, Switzerland) and LEICA DFC Twain software v. 4.1 (Leica, Germany). From each animal, 4–6 sections were analyzed, and 2–3 images from each section were included and averaged to obtain a representative value.

### 2.6. Preparation of Crude Synaptosomal P2 Fraction

Six animals were used for P2 synaptosomal preparation in both the Cont and TMT+cTBS groups. In the TMT group, two animals did not survive the intoxication protocol, resulting in four samples available for biochemical analyses; the remaining brains were used for immunohistochemical analyses. This clarification has been included to avoid confusion regarding group sizes.

First, animals (Cont = 5, TMT = 4, TMT + cTBS = 6) were decapitated (Harvard Apparatus, Holliston, MA, USA), brains were removed, and hippocampi were dissected for preparation of crude synaptosomal fractions (P2). Tissue samples were placed in 10 volumes of ice-cold 0.32 M sucrose in 5 mM Tris-HCl buffer, pH 7.4 (1 g of wet tissue/10 mL of buffer), and homogenized manually in a Teflon/glass homogenizer (0.20 mm spacing) by approximately 20 gentle up and down movements. Crude nuclear fraction and cell debris were removed by centrifugation twice at 1000× *g* for 10 min. The resulting supernatant was transferred and centrifuged at 10,000× *g* for 20 min to obtain the crude synaptosomal pellet, which was resuspended in 5 mM Tris-HCl, pH 7.4. The isolated crude synaptosomal P2 fraction contains the majority of synaptosomes and free mitochondria, as well as other membrane fragments derived from glial cells and neurons [47]. After isolation, protein concentration was determined using the Pierce^TM^ BCA Protein Assay Kit (Thermo Scientific, Waltham, MA, USA) according to the manufacturer’s instructions.

### 2.7. Western Blot Analysis

Western blot analysis was performed using the previously prepared hippocampal P2 fraction. Each sample, containing the same amount of total protein, was diluted in Laemmli sample buffer (250 mM Tris-HCl, pH 6.8, 10% SDS, 30% glycerol, 5% β-mercaptoethanol, 0.02% bromophenol blue), separated on an 8% SDS-PAGE gel, and transferred to PVDF membranes (Imobilon-P membrane, Millipore, Burlington, MA, USA). The membranes were blocked for 1 h in TBS containing 5% non-fat milk (Sigma-Aldrich, St. Louis, MO, USA) and 0.1% Tween 20 (Sigma-Aldrich, USA), then incubated overnight at 4 °C with the appropriate primary antibodies (Table 1). The next day, the membranes were incubated for 1 h with the corresponding horseradish peroxidase-conjugated secondary antibodies (Table 1). After washing in TBST, the membranes were incubated with the enhanced chemiluminescent system (Immobilon Western Chemiluminescent HRP Substrate, Millipore, USA), and the immunoreactive bands were detected on X-ray films in the darkroom, scanned, and saved in .tiff format. β-Actin was used as a loading control. Signal intensity was evaluated using ImageJv. 1.53t software. The analysis was performed at least three times for each target protein to ensure the reliability of the results. Molecular weight ladders (PageRuler™ Plus Prestained Protein Ladder, Thermo Fisher Scientific) were used in all Western blot runs to verify that the observed bands corresponded to the expected molecular weights of the target proteins.

### 2.8. Statistical Analysis

All data were analyzed for normality using the Shapiro–Wilk test, and appropriate parametric or nonparametric tests were applied. Behavioral data and immunoblot results were analyzed using a one-way ANOVA test, followed by Tukey’s post hoc test. The micrograph’s signal intensity was analyzed using the unpaired, two-tailed Student’s *t*-test. Values of *p* < 0.05 were considered statistically significant. Data analysis and graphical representation were performed using the GraphPad Prism 9.0 software package (San Diego, CA, USA).

## 3. Results

### 3.1. cTBS Treatment Decreases Anxiety-like Behavior and Improves Learning and Memory Following TMT-Induced Hippocampal Neurodegeneration

**Figure 1:**
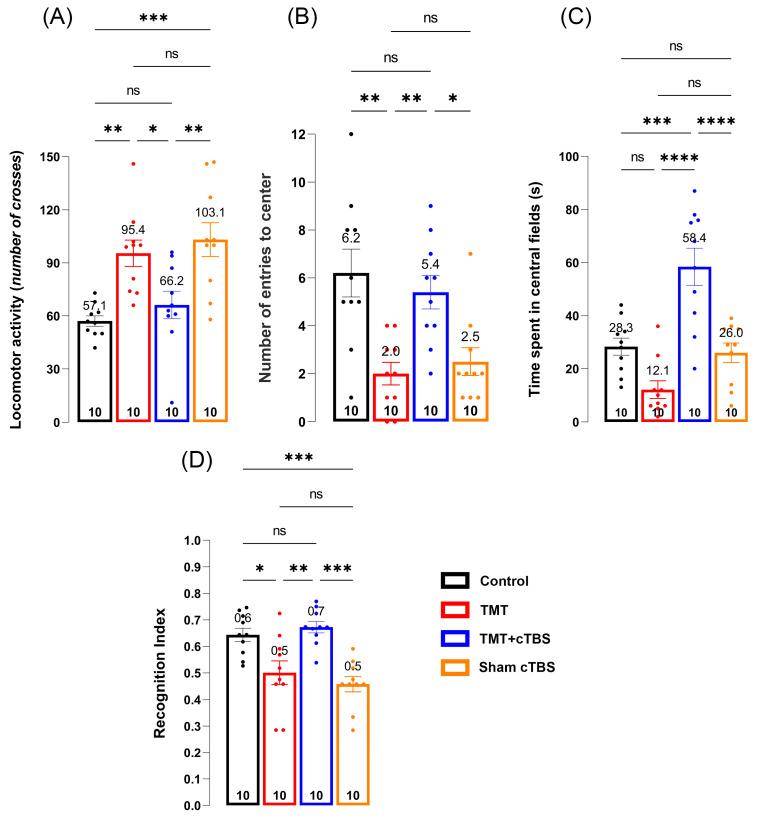
Effects of cTBS treatment on anxiety-like behavior, learning, and memory following TMT-induced neurodegeneration. Results of the open field test—(**A**) locomotor activity calculated by total number of crosses, (**B**) number of entries to the center, (**C**) time spent in center [s], and novel object recognition test—(**D**) recognition index. Data were statistically analyzed by one-way ANOVA, followed by Tukey’s post hoc test (dots in the graphs represent values of individual animals). Symbols indicate significant differences between groups: * *p* < 0.05, ** *p* < 0.01, *** *p* < 0.001, **** *p* < 0.0001, ns—no significance, number of animals *per* group: 10.

The open field test was used to assess the effects of cTBS on TMT-induced hyperactivity and anxiety-like behavior. Significant changes were observed in anxiety-like behavior as expressed by the locomotor/exploratory activity (Figure 1A, F_3;36_ = 9.142, *p < 0.001*), number of center entries (Figure 1B, F_3;36_ = 8.486, *p = 0.0002*), and time spent in the central quadrants of the arena (Figure 1C, F_3;36_ = 18.02, *p < 0.0001*). As expected, both the TMT and Sham groups showed significantly higher locomotor activity compared with the control group (*p = 0.004* and *p = 0.0005*, respectively), whereas cTBS treatment returned activity to levels comparable with the control group (*p = 0.8179*). Although animals in the TMT and Sham groups showed increased locomotor activity, this was limited to the peripheral quadrants, and they very rarely entered (*p = 0.0011* and *p = 0.0043*) or spent time in the central areas (*p = 0.0003* and *p* = 0.0001) compared to the control. Animals treated with cTBS entered the central areas similarly to those in the control group (*p = 0.8584*), but the time spent in the central areas was significantly higher after cTBS compared with all groups studied (control: *p = 0.0003*, TMT: *p < 0.0001*, Sham cTBS: *p < 0.0001*).

The effects of cTBS treatment on TMT-induced learning and memory impairments were examined using NORT. A significant effect of cTBS was observed in hippocampus-dependent learning and memory (Figure 1D, F_3;36_ = 11.43, *p < 0.0001*). In the test phase, animals from the TMT and Sham cTBS groups had an RI significantly lower than the control (*p = 0.0149* and *p = 0.0010*, respectively), indicating a lack of ability to discriminate between novel and familiar objects. Prolonged cTBS treatment resulted in an increase in the RI compared to the control level (*p = 0.9877*), suggesting a significant improvement in learning and memory deficits in cTBS-treated animals after TMT intoxication (TMT: *p = 0.0024*, Sham cTBS: *p = 0.0001*).

### 3.2. cTBS Treatment Reduced Reactive Microgliosis in CA1 and Medial/Proximal CA3 Regions Following TMT-Induced Hippocampal Neurodegeneration

**Figure 2:**
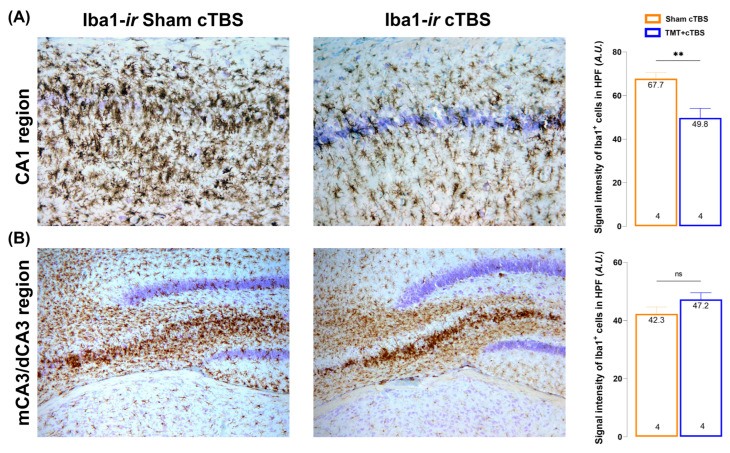
Histological examination and signal intensity measurement of Iba1 immunoreactivity in the CA1 region of the hippocampus (**A**) and in medial CA3(mCA3)/proximal CA3 (pCA3) (**B**). Data were analyzed using the unpaired, two-tailed Student’s *t*-test. Symbols indicate significant differences between sham cTBS and TMT + cTBS groups: ** *p* < 0.01, ns—no significance, number of animals *per* group: 4. The supporting information can be downloaded at Supplementary Material.

Trimethyltin is known to selectively disrupt CA1/CA3 pyramidal neurons, leading to behavioral changes. The observed improvements at the behavioral level, therefore, prompted us to examine the hippocampus of animals treated with cTBS and Sham histologically using the Iba1 marker. As described previously, TMT-induced severe hippocampal degeneration of the CA1/CA3 region was reflected by numerous highly reactive Iba1^+^ cells covering the entire CA1/CA3 region and invading the granular cell layer of the dentate gyrus (Figure 2A,B). After cTBS treatment, Iba1^+^ cells still exhibited a reactive phenotype, but the signal intensity in the CA1 region of stimulated animals was significantly lower (Figure 2A, *p* < 0.01) compared to Sham. Iba1 signal intensity was not changed after cTBS in CA3 (Figure 2B, *p = 0.1519*), but the absence of Iba1 from granular cells of the dentate gyrus was conspicuous (Figure 2B).

### 3.3. cTBS Treatment Modulated the Protein Expression of NMDAR Subunits and vGluT1 in HIP of TMT-Treated Animals

**Figure 3:**
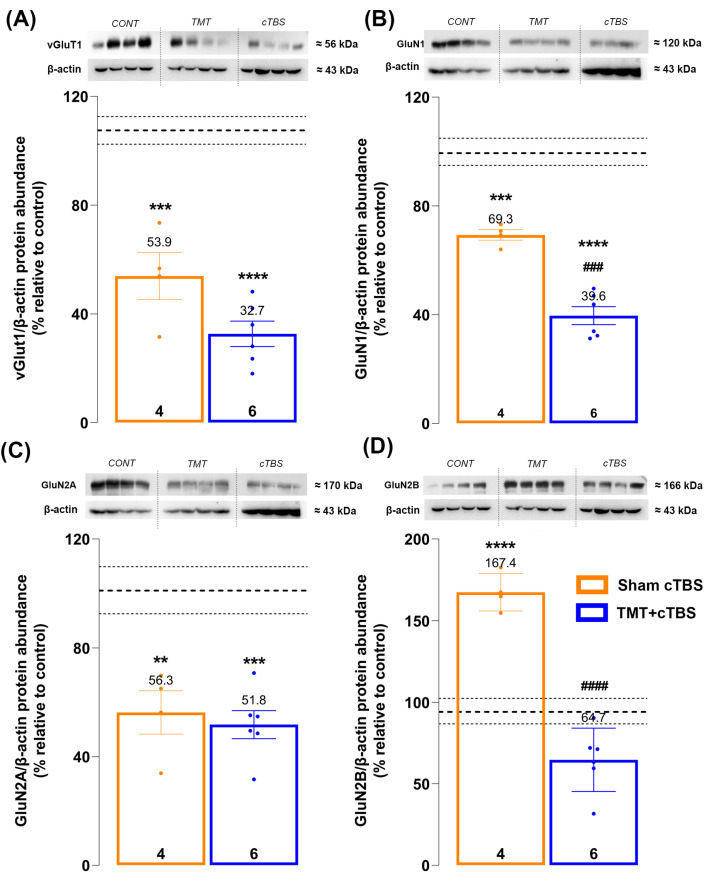
Representative immunoblots and quantitative data of Western blot analysis of target proteins expression in hippocampal P2 fraction: (**A**) vGlut1, (**B**) NR1, (**C**) NR2A, (**D**) NR2B. Data were statistically analyzed by one-way ANOVA, followed by Tukey’s post hoc test, and expressed as a percentage of the mean values of the CONT group ± SEM (dots in the graphs represent values of individual animals). The control group is shown in the representative blot images above the graphs but was not included as a separate bar in the graphs to maintain clarity in the main comparisons. Symbols indicate significant differences compared to control: ** *p* < 0.01, *** *p* < 0.001, **** *p* < 0.0001, or Sham cTB. S group: ^###^ *p* < 0.001, ^####^ *p* < 0.0001, number of animals *per* group: 4–6. The supporting information can be downloaded at Supplementary Material.

Both treatments induced significant changes in the levels of all investigated proteins (vGluT1: F_2,12_ = 45.39, *p* < *0.0001*; NR1: F_2,12_ = 68.08, *p < 0.0001*; NR2A: F_2,12_ = 14.67, *p = 0.0006*; NR2B: F_2,11_ = 51.34, *p < 0.0001*; Figure 3A–D, respectively). Trimethyltin treatment resulted in a significant decrease in the levels of vGluT1 (Figure 3A, *p = 0.0002*) as well as NR1 (Figure 3B, *p = 0.0005*) and NR2A (Figure 3C, *p = 0.0035*) subunits compared with the control. Unexpectedly, TMT intoxication triggered a distinct increase in NR2B levels compared with the control (Figure 3D, *p < 0.0001*). Regarding the effects of cTBS, a significant decrease in NR1 levels was detected compared with the TMT group (Figure 3B, *p* = 0.0004), indicating a more prominent reduction in the total number of remaining NMDARs. Moreover, cTBS treatment restored NR2B levels to those of the control group (Figure 3D, *p < 0.0001*), possibly alleviating the deleterious signaling mediated by NR2B-containing NMDARs in TMT-treated animals. However, cTBS treatment exerted no statistically significant effect on the levels of vGlut1 and NR2A compared with the TMT group (Figure 3A,C; *p = 0.0670* and *p = 0.9027*, respectively), although a trend towards a decrease can be observed for vGlut1.

## 4. Discussion

The present study is the first to demonstrate the beneficial effects of cTBS treatment on different pathological aspects of TMT-induced neurodegeneration, already established as an Alzheimer’s-like disease model, and to suggest a possible neuroprotective mechanism of cTBS action, partially mediated by modulation of protein expression of hippocampal NMDAR subunits. As previously mentioned, acute TMT intoxication is a well-characterized model of hippocampal neurodegeneration and associated limbic and cortical regions, producing learning and memory deficits as well as behavioral changes such as aggression, increased anxiety-like behavior and hyperlocomotion [15,16,17], which were also confirmed in this study. The applied cTBS treatment successfully improved parameters indicative of increased anxiety-like behavior and alleviated learning and memory deficits following TMT intoxication. The observed improvements may be a direct consequence of cTBS-induced LTD but may also result from modulation of key triggers of the neurodegenerative cascade, such as NMDAR. Regarding anxiety and cTBS effects, the literature reports potential improvement of psychological symptoms in generalized anxiety disorder [48] and anxiety associated with obsessive–compulsive disorder [49]. Beneficial effects of cTBS on various aspects of learning and memory have been demonstrated in several studies, including improvements in learning and memory in a mouse photochromic model of stroke [50], interference with the formation of aversive memories in humans [51], improved decision-making through reduced impulsivity in healthy volunteers [52,53,54], and enhancement of other executive functions [54,55].

From a molecular perspective, glutamatergic neurotransmission, particularly the NMDAR complex, is a well-characterized cornerstone of hippocampal-dependent learning and memory, as well as anxiety behavior [12,55]. Two of the three NMDAR subunits studied (NR1 and NR2A), as well as vGluT1, were decreased in the TMT group compared with the control, which may seem unexpected given the hyperexcitability associated with TMT intoxication. However, it should be kept in mind that the overall reduction in the number of NMDARs in hippocampus is a direct consequence of TMT-induced neuronal loss developing over three weeks, as mentioned previously [24]. TMT intoxication triggers two major molecular events that contribute to neuronal loss: (1) NMDAR-mediated excitotoxicity and (2) massive microgliosis [17]. This study focused primarily on the first outcome, NMDAR-mediated excitotoxicity, and showed that NR1 and NR2B levels decreased significantly after cTBS treatment compared to the TMT group, while levels of NR2A and vGluT1 remained unchanged. The decrease in NR1 protein levels, a constitutively expressed NMDAR subunit [56], may indicate a further cTBS-induced reduction in the total number of remaining NMDARs. It is possible that this specific effect limits the extent of initial and/or delayed NMDAR-mediated excitotoxicity, bearing in mind the cumulative effects of prolonged exposure to cTBS. In the context of TMT intoxication, the most notable finding is the significant increase in NR2B levels. Given the reduction in NR1 subunits following TMT, which may indicate a reduced total number of NMDARs, and the loss of hippocampal neurons in the TMT group, a rise in any other NMDAR subunit is rather unexpected. This may suggest that the remaining population of glutamatergic neurons is “NR2B-enriched” and could be exacerbating the ongoing neurodegenerative cascade through NR2B-mediated death signaling pathways [57]. As shown, cTBS treatment significantly decreased NR2B levels, returning them to control values and potentially reducing deleterious signaling mediated by NR2B-containing NMDARs. This finding may be promising for potential AD therapy, especially considering that NR2B NMDARs are among the most important targets in AD treatment [58]. Memantine, a non-competitive NR2B NMDAR antagonist [59], is one of the six drugs approved by the US Food and Drug Administration for the symptomatic treatment of AD [60], while other NR2B NMDAR antagonists have shown positive effects in various animal models of AD [61,62]. Evidence for potential cTBS-mediated amelioration of glutamate excitotoxicity is also found in recent work by Cember and colleagues (2022), who reported decreased glutamate concentration at the site of cTBS action in healthy volunteers [63]. It should be noted that although the vGluT1 level remained statistically unchanged after the cTBS treatment, the trend towards a decrease is quite evident, which is partly consistent with previous studies reporting a decrement in vGluT1 [64,65].

As previously mentioned, TMT triggers massive microglial overactivation in hippocampus, which was also confirmed in this study. It is well known that reactive gliosis and neuroinflammation are histological hallmarks of AD and that microglial activation is observed in both the brains of AD patients and animal models [66,67,68]. Interestingly, although the reactive phenotype of Iba1+ cells remained unchanged following cTBS treatment, the markedly reduced Iba-1 signal intensity in the CA1 region suggests a possible reduction in microglial activity. However, it remains unclear whether the potentially reduced microgliosis is a cause or a consequence of reduced neuronal death and alleviated NMDAR excitotoxicity. To date, there is very little data on the effects of cTBS on microglial activity and neuroinflammation. The most important data come from Wu and colleagues (2020), who showed that cTBS significantly reduced the extent of post-ischemic inflammatory activation in a rat stroke model. They demonstrated that cTBS may mediate a protective effect, as evidenced by increased M2 polarization and decreased density of Iba1+ and GFAP+ positive cells after treatment [69].

Finally, the authors would like to briefly discuss the effects of the opposite excitatory TBS protocol, iTBS, in this model and other AD-like models. Namely, the authors have previously shown positive effects of iTBS on TMT-induced behavioral, learning, and memory impairments, possibly due to the rescue of PI3K/Akt/mTOR in the same trimethyltin-induced AD-like model [16]. Other studies using different variants of AD-like models have also demonstrated similar results [70,71]. This raises a logical question: how can protocols that produce opposite effects on neuronal excitability both have positive effects in the same AD-like animal model? A partial explanation for this phenomenon may be found in the comprehensive work of Funke and colleagues (2013), who investigated dose-dependent changes in several proteins after both iTBS and cTBS. They demonstrated that various proteins, such as parvalbumin, calbindin, glutamic acid decarboxylase 65 and 67, c-Fos, and others, exhibit the same pattern of change after both cTBS and iTBS, and that marked differences may occur when comparing specific blocks of stimulation or the difference from the effect of a previous block [65]. Furthermore, it is possible that iTBS and cTBS exert similar effects on behavior, memory, and learning via different molecular pathways. This is supported by the study by Lee et al. (2023), which showed that both iTBS and cTBS can improve depressive-like behavior in a rat model of treatment-resistant depression. They found that impaired LTP and LTD were improved by iTBS, while cTBS treatment improved aberrant LTD, suggesting that the synaptic function modulated by these two opposing rTMS paradigms may be mediated by different molecular mechanisms [72]. In view of this, our future scientific efforts will focus on the comprehensive investigation of the molecular changes underlying the effects of both cTBS and iTBS.

### Limitations of the Study

**Animal model**. The authors reiterate that the model presented here mimics some aspects of AD, such as altered behavior, learning and memory deficits, microgliosis, neuronal death, and so on. The authors’ future research will include examination of cTBS on another AD model, as well as investigation of the molecular mechanisms of cTBS actions in healthy animals.

**TMS device**. It is extremely important to discuss the technical limitations of the MagStim Rapid2 device, as the characteristics of the coil, mainly its size but also manual placement, influence the results of the study and the discussion of the mechanism of cTBS effects. More specifically, the observed effects of cTBS result from stimulation of both cortical and subcortical structures and their connections, as well as glial cells, so they cannot simply be attributed to enhancement of cortical LTD.

## 5. Conclusions

In summary, for the first time, the beneficial effects of cTBS on learning, memory, and behavioral alterations have been reported alongside modulation of hippocampal NMDAR subunits in a model of neurodegeneration that mimics some major aspects of AD pathology. The cTBS effect on the levels of the studied proteins may represent part of a neuroprotective cascade responsible for the observed behavioral, learning, and memory improvements and microglia-related amelioration in the CA1 region. However, the authors’ ongoing comprehensive study of cTBS effects will lead to a more detailed understanding of its mechanisms of action and therapeutic capacity in the treatment of neurodegenerative diseases, including AD.

## Figures and Tables

**Table 1:** List of primary and secondary antibodies used in the study.

Antigen	Manufacturer and Catalog No	Species and Dilution
Iba1	Abcam, UK, ab5076 RRID: AB_2224402	Goat polyclonal; 1:400
NR1	Cell Signaling Technology, Danvers, MA, USA, #5704 RRID: AB_1904067	rabbit monoclonal; 1:1000
NR2A	Merck Millipore, Burlington, MA, USA, #07-632 RRID: AB_310837	rabbit monoclonal; 1:1000
NR2B	Abcam, UK, 93610 RRID: AB_10561972	mouse monoclonal; 1:4000
vGluT1	Abcam, UK, 134283 RRID: AB_2923539	mouse monoclonal; 1:4000
β-actin	Thermo Fisher Scientific, Waltham, MA, USA; PA1-21167 RRID: AB_557422	rabbit polyclonal; 1:5000
mouse IgG	R&D systems, bio-techne, Minneapolis, MN, USA; HAF007 RRID: AB_357234	goat polyclonal; 10,000
rabbit IgG	Invitrogen, Carlsbad, CA, USA; # 31460 RRID: AB_228341	goat polyclonal; 1:10,000

## Data Availability

The original contributions presented in this study are included in the article/Supplementary Material. Further inquiries can be directed to the corresponding author.

## Supplementary Materials

The following supporting information can be downloaded at https://www.mdpi.com/article/10.3390/biomedicines14020391/s1, Figure S1: original, unadjusted micrographs and original, unprocessed, and uncropped blot images.
